# Synthesis of Polypeptides and Poly(α-hydroxy esters) from Aldehydes Using Strecker Synthesis

**DOI:** 10.1021/acsomega.3c04870

**Published:** 2023-10-18

**Authors:** Ester Abtew, Abraham J. Domb

**Affiliations:** The Alex Grass Center for Drug Design & Synthesis and the Center for Cannabis Research, School of Pharmacy, Institute of Drug Research, Faculty of Medicine, The Hebrew University of Jerusalem, Jerusalem 9112001, Israel

## Abstract

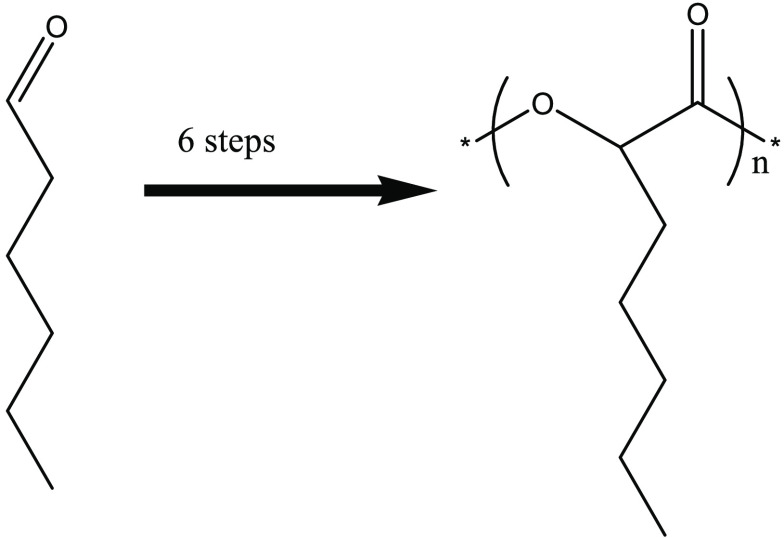

This report presents a versatile approach for the synthesis of new polypeptide and polyester-based biomaterials. The well-established Strecker reaction was utilized, with hexanal serving as the model aldehyde, to synthesize α-amino and α-hydroxy acids as monomer units for the polymer system. Following the formation of the corresponding amino and hydroxy acid monomers, they were subsequently converted to *N*-carboxy and *O*-carboxy-anhydrides. The resultant cyclic anhydride molecules were then polymerized via ring-opening polymerization to yield the corresponding polypeptides and polyesters. This report establishes a straightforward methodology for the synthesis of new polypeptide and poly(*a*-hydroxy acid)-based biomaterials, thereby expanding the existing library of polymers for various biomedical applications.

## Introduction

Synthetic polymers have a broad range of biomedical applications, including use as absorbable sutures, orthopedic implants, and drug delivery carriers. This is because of their mechanical and physical properties that can be tailored to fit specific requirements such as biodegradability, mechanical strength, flexibility, solubility, and thermal properties.^1,2^ Peptides derived from α-amino acids and polyesters derived from α-hydroxy acids are among the most commonly used synthetic biopolymers.^3^ These biopolymers have been investigated for a variety of biomedical applications including use as drug delivery carriers and tissue engineering scaffolds.^3,4^ Notably, aliphatic polyesters derived from α-hydroxy acids such as lactide and glycolide have generated significant interest owing to their long-standing safety record. They degrade in vivo into their acid counterparts that further metabolize to H_2_O and CO_2_. These polymers exhibit remarkable strength and high-modulus thermoplasticity, and they can be easily processed using conventional processing techniques.^5,6^

Synthetic polypeptides have been mainly synthesized from natural α-amino acids.^7,8^ Polypeptides are poly(amino acid) linked by peptide bonds. They exhibit exceptional biodegradability and biocompatibility, making them versatile synthetic polymers with structure-mimicking natural proteins. As a result, polypeptides possess unique properties suitable for various biomedical applications. Notably, they can self-assemble into well-defined three-dimensional (3D) structures, especially desirable when hierarchical architecture or complex functionalities are needed for various biomedical applications.^8,9^ These synthetic polypeptides may be homo- or copolymers of amino acids.^10^

However, the variability of hydroxy acid-based polyesters and amino acid-based polypeptides is limited, likely due to the limited availability of these monomers from natural sources and challenges in their synthesis. Therefore, new aliphatic amino acids and hydroxy acids, along with their derivatives bearing new functional groups, would be a valuable extension to the existing library of biodegradable polymers. These functional groups can also be further derivatized to yield biodegradable polymeric prodrugs,^11^ conjugates, and potential candidates for use as scaffolds in tissue engineering by cross-linking the polymer chains with these functional groups. In recent years, there has been an intensive effort to expand the versatility of “PLA-like” polyesters and polypeptides by synthesizing new derivatives of α-hydroxy acids and α-amino acids.^12−14^ Some research groups have synthesized new classes of polyesters from hydroxylated amino acids.^1,15−22^ For instance, Deng et al. employed lignocellulosic biomass-derived α-hydroxyl acids to produce α-amino acids, including alanine, leucine, valine, aspartic acid, and phenylalanine employing Ruthenium nanoparticles supported on carbon nanotubes (Ru/CNT) as catalyst.^23^ Initially, these hydroxylated amino acids were synthesized from nonpolar amino acids such as phenylalanine and polymerized into a new class of aliphatic polyesters. Later, this concept was extended to amino acids with side group functionality such as lysine, serine, and tyrosine, resulting in water-soluble functional polyesters. Lately, our group synthesized a new family of functional “PLA-like” polypeptides and polyesters with acetonide or benzyl ether-protected monosaccharide repeating groups.^24,25^ For polymerization, we used saccharide monomers with amino acid functionality (glucose amino acid) or we functionalized other saccharides to the correspondent α-amino acid and α-hydroxy acid before polymerization. The protecting groups were cleaved after polymerization to obtain a new family of water-soluble poly(glycol-peptides) and poly(glycol-esters) with pendant hydroxyl groups. These hydroxy acids and amino acids can also be produced from natural resources, but they have restricted variability. Therefore, there is a need to expand the library of available hydroxyl acids and amino acids, to enhance the versatility and functionality of these essential biodegradable polymers. Expanding the library of α-hydroxyl acids and α-amino acids will make it possible to synthesize new poly(α-amino acids) and poly(α-hydroxy acids) with high variability and functionality.

We chose the Strecker amino acid synthesis method, which is a well-established approach for the preparation of α-amino acids from aldehydes. The Strecker synthesis involves the simultaneous reaction of ammonia and hydrogen cyanide with the aldehyde, followed by hydrolysis of the resulting amino nitrile to amino acid.^26−29^ We chose the Strecker reaction for the synthesis of α-amino acids and α*-*hydroxyl acids for several reasons. First, this methodology is simple and cost-effective for the preparation of these monomers for both laboratory and industrial use.^30^ Second, it can be applied to all aldehydes. Since this method is universal to aldehydes, we can also apply it to monosaccharides that also have an aldehyde functional group. This will enable the synthesis of new glycol-α-amino and hydroxyl acid derivatives. It will also expand the library of new glycol (polypeptides) and glyco (polyesters) already synthesized in our previous work. The first goal was to synthesize a new family of aliphatic polypeptides and polyesters from aliphatic aldehydes by using the Strecker method. Hexanal was used as a model aldehyde for the synthesis of aliphatic α-amino acids and α-hydroxy acids, which were subsequently polymerized to yield new derivatives of aliphatic polypeptides and polyesters. By doing so, we aimed to further expand the library of the poly(glycol-peptide)s and poly(glycol-ester)s synthesized in our previous work.

## Materials

Isobutyl alcohol (IB), *n*-hexanal, trimethylsilyl cyanide (TMSCN), ZnI_2_, tetraethylene glycol, KF, NH_4_COO, palladium/charcoal activated (10% Pd), triethylamine, 4-dimethylamino pyridine (DMAP), H_2_O_2_ (30% w/w in H_2_O), K_2_CO_3_, DMSO, NaOH, triphosgene, hexamethyldisilazane (HMDS), and other fine chemicals were purchased from Sigma-Aldrich (Rosh Ha’ayin, Israel), and BioLab (Jerusalem, Israel) and used without further purification. All solvents used were of analytical grade and were freshly distilled before use.

### Spectroscopic Measurements

^1^H and ^13^C NMR spectra (CDCl_3_) were obtained on a Varian 300 or 500 MHz NMR spectrometer (Varian, Inc., Palo Alto, CA) in 5 mm tubes. Depending on solubility, CDCl_3_, DMSO (*d*_6_), or D_2_O were used as solvents. Electrospray ionization mass spectrometry (ESI MS) was recorded on a ThermoQuest, Finnigan LCQ-Duo instrument in positive or negative ionization mode (whichever is appropriate depending on the compound). Fourier transform infrared (FT-IR) spectroscopy analysis was performed using a Smart iTR ATR sampling accessory for Nicolet iS10 spectrometer with a diamond crystal (Thermo Scientific, Massachusetts).

### Molecular Weight Determination

The molecular weight of hydrophobic polymers was determined by a Gel Permeation Chromatography (GPC) system, Waters 1515. An isocratic HPLC pump with a Waters 2410 refractive index detector, a Waters 717 plus auto sampler, and a Rheodyne (Cotati, CA) injection valve with a 20 μL-loop were used. The samples were eluted with CHCl_3_ (HPLC grade) through linear Styragel HR3 column (Waters) at a flow rate of 1 mL/min. Molecular weights were determined relative to polystyrene standards (Polyscience, Warrington, PA) with a molecular weight range of 500–30,000 and GPC column.

### DSC Measurements

Samples (5 mg) were weighed by using a microanalytical balance. The thermal behavior of the polymers was investigated using a DSC Q4000 (TA Instruments, New Castle, Deleware). DSC thermograms were recorded by gradually heating from −40 to 400 °C at a rate of 10 °C min^–1^. A preheating and cooling cycle from 25 to 100 °C was performed before measuring the actual samples.

### Synthesis

All reactions were conducted in oven-dried glassware under a dry N_2_ atmosphere using dry solvents. Dichloromethane (DCM) was distilled and collected over 4 Å molecular sieves. Ethyl acetate was freshly distilled from Calcium hydride, and tetrahydrofuran was dried by distillation from sodium ribbons. Deionized water with a resistivity of 18 MΩ·cm was obtained using a Millipore Milli-Q Biocel A10 purification unit. All other commercially obtained reagents were used as received. Thin-layer chromatography (TLC) was conducted with Merck gel 60 F254 precoated plates (0.25 mm) on aluminum sheets and visualized using a combination of ultraviolet (UV), Iodine chamber (mixture of molecular I_2_ and silica), and sulfuric acid charring (20% concentrated H_2_SO_4_ in anhydrous methanol). Column chromatography was performed on silica gel with particle sizes of 100–200 or 60–120 mesh.

#### Synthesis of Polypeptide from Hexanal

##### Synthesis of 2-(Benzylamino)heptanenitrile (**2**)

A round-bottom flask containing 2 g (20 mmol) of hexanal and a catalytic amount of ZnI_2_ was purged with N_2_. Trimethylsilyl cyanide (TMSCN) (2 mL) was added to this mixture and stirred for 15 min. Then, 2 mL of benzyl amine was added, and the reaction continued overnight. TLC (30% ethyl acetate in hexane) showed that the entire starting material had been consumed. The resulting material was purified by column chromatography on silica gel by using the same solvent system used for TLC. ^1^H NMR (300 MHz, cdcl_3_) δ 7.71–7.15 (m, 5H), 4.08 (d, *J* = 12.9 Hz, 1H), 3.83 (d, *J* = 12.9 Hz, 1H), 3.50 (t, *J* = 7.1 Hz, 1H), 1.77 (q, *J* = 7.4 Hz, 2H), 1.31 (dt, *J* = 6.2, 3.0 Hz, 6H), 0.90 (t, *J* = 6.7 Hz, 3H). ^13^C NMR (75 MHz, DMSO-*d*_6_) δ: 139.85, 128.51, 127.29, 121.14, 51.16, 49.80, 33.00, 31.14, 31.12, 25.39, 22.42, 14.24. ESI MS (+ve ionization): calculation for C_14_H_20_N_2_ 216.32. However, the observed is 191.11 (M + H). This *M*_w_ is appropriate for the monomer with eliminated nitrile group FTIR: (neat) [cm^–1^], 2223(−C≡N_nitril_). Yield 85%

##### Synthesis of 2-(Benzylamino)heptanamide (**3**)

1.9 g of the oily amino nitrile (compound **2**) was dissolved in DMSO (20 mL) and treated with an excess amount of K_2_CO_3_. Aqueous H_2_O_2_ (50 mL) was added to the mixture over 30 min, keeping the temperature below 5 °C. After the addition of the H_2_O_2_, the temperature was kept at 10–15 °C overnight. The resulting amino amide product was precipitated out and filtered through Buchner. The white precipitate was dried by freeze-drying to obtain compound **3** in a yield of 70%. ^1^H NMR (300 MHz, Chloroform-*d*) δ 7.46–7.17 (m, 5H), 7.08 (s, 2H), 5.72–5.33 (m, 1H), 3.82 (d, *J* = 13.1 Hz, 1H), 3.69 (d, *J* = 13.1 Hz, 1H), 3.14 (dd, *J* = 7.6, 5.1 Hz, 1H), 1841.47q 2H 1.39–1.17 (m, 6H), 0.86 (t, *J* = 6.8 Hz, 3H). ^13^C NMR (75 MHz, DMSO-*d*_6_) δ: 177.55, 139.9, 128.39, 128.05,127.30, 62.50, 52.87, 33.64, 31.60, 25.54, 22.47, 14.00,. ESI MS (+ve ionization): calcd for C_14_H_22_N_2_0 234.17 observed 235.12 (M + H). FTIR: (neat) [cm^–1^] 1672, 1609 (−C=O, amide), yield 70%.

##### Synthesis of 2-(Benzylamino)heptanoic acid (**4**)

Alkaline hydrolysis of the amide to carboxylic acid: The hydrolysis of the amide to carboxylic acid was done in an ethanol/water mixture using an excess amount of NaOH (under reflux 80 °C) overnight. When most of the starting material had been consumed, ethanol was evaporated under reduced pressure. Then, the resulting aqueous solution was washed (×3) with ether to remove the residual starting material (amide). The aqueous solution was acidified using concentrated HCl. At pH ∼5, the product precipitated out and filtered through a vacuum Buchner. It was washed several times with cold water. The resulting white material was dried under freeze-drying to obtain a crude white powder. ^1^H NMR (300 MHz, DMSO-*d*_6_) δ 12.11 (s, 1H), 8.25–6.94 (m, 5H), 3.82 (s, 2H), 2.49 (t, 1H), f1.36(q, 2H)1.23 (m, 6H), 0.83 (td, *J* = 6.7, 3.5 Hz, 3H). ESI MS (+ve ionization): calcd for C_14_H_22_NO_2_ 235.32 observed 236.25 (M + H). FTIR: (neat) [cm^–1^] 1710(–C=O, acid), yield 40%.

##### Synthesis of 2-Aminoheptanoic acid (**5**)

Benzyl deprotection of amine was performed by hydrogenolysis using ammonium formate as a hydrogen-donating agent and palladium on charcoal (10%) as a catalyst as described by Ram et al.^2^ Anhydrous ammonium formate (5 mmol) was added in a single portion under nitrogen to a stirred suspension of 2-(benzylamino)heptanoic acid (230 mg, 1 mmol) and an equal weight of 10% Pd–C in dry methanol (50 mL). The resulting reaction mixture was stirred for 6 h at reflux temperature, and the reaction was monitored by TLC. The catalyst was removed by filtration after completion of the reaction. The resulting powder was concentrated by using a rotary evaporator. Hest et al. also reported the preparation of 2-aminoheptanoic acid by alkylation of diethyl acetamidomalonate with the appropriate tosylate, followed by hydrolysis.^31^^1^H NMR showed full elimination of the benzyl proton. ^1^H NMR (300 MHz, DMSO-*d*_6_) δ 12.43 (s, 1H), 3.46 (t, *J* = 4.7 Hz, 1H), 1.61–1.41 (q, 2H)1.23 (m, 6H), 1.41–0.99 (m, 6H), 0.92–0.54 (t, 3H).ESI MS (negative ionization): calcd for C_7_H_15_NO_2_ 145 observed 144.03 (M – H). Yield 70%.

##### Synthesis of 4-Pentyloxazolidine-2,5-dione (**6**)

A 250 mL dry round-bottom flask was charged with compound **5** (150 mg, 1 mmol) and triphosgene (276 mg, 1 mmol) under argon. Then, dry THF (20 mL) was added, and the reaction mixture was stirred at 55 °C for 3 h. The clear reaction mixture was then concentrated under a vacuum, followed by precipitation in hexane. ^1^H NMR (300 MHz, Chloroform-*d*) δ 6.19 (s, 1H), 4.35 (q, *J* = 6.8 Hz, 1H), 2.11–1.63 (m, 2H), 1.56–1.07 (m, 6H) 0.89 (d, *J* = 6.7 Hz, 3H). ^13^C NMR (75 MHz, Chloroform-*d*) δ: 169.45, 109.99, 57.63, 31.73, 31.04, 24.38, 22.27, 13.86. FT-IR: (neat) [cm^–1^] 1790 and 1820 (–C=O, oxazolidine-2,5-dione). Yield 80%.

##### Ring-Opening Polymerization Reaction (**7**)

For polymerization, 4-pentyloxazolidine-2,5-dione (**6**) was dissolved in 2 mL of dry DMF and purged with N_2_ atmosphere. Next, hexamethyldisilazane (HMDS) was added to the reaction mixture in a ratio of 1:100 (initiator/monomer). The reaction was continued for 24 h by purging constantly with N_2_ to remove the CO_2_ evolving from the ROP reaction. The polymer was precipitated by adding excess methanol. The resultant polymer was collected after centrifuge, and the residual solvent was removed by adding water and freeze-dried.

#### Synthesis of Polyester from Hexanal

##### Synthesis of 2-((Trimethylsilyl)oxy)heptanenitrile (**8**)

Hexanal (2 g) was stirred with a catalytic amount of ZnI_2_ under inert atmosphere for 5 min. 2.5 mL of TMSCN was added, and the reaction continued for 2 h. TLC showed that the entire starting material had been consumed.

##### Synthesis of 2-Hydroxyheptanenitrile (**9**)

PEG 200 and KF were added and purged with N_2_ to cleave the silyl ether into the mixture from the procedure above. This reaction continued overnight. TLC (ethyl acetate/hexane) (20:80) showed that the entire starting material had been consumed. Then, the reaction mixture was suspended in water, extracted with ethyl ether, and concentrated using a rotary evaporator. The resulting pasty material was purified using silica gel column chromatography, solvent system (ethyl acetate/hexane) (20:80). ^1^H NMR (300 MHz,) δ 4.45 (t, *J* = 6.8 Hz, 1H), 3.32 (s, 1H), 1.82 (q, *J* = 7.2 Hz, 2H), 1.53–1.41 (m, 2H), 1.31 (td, *J* = 9.1, 8.2, 4.6 Hz, 4H), 0.89 (t, *J* = 6.5 Hz, 3H). ^13^C NMR (300 MHz, Chloroform-*d*) δ: 120.10, 61.29, 35.09, 31.04, 24.20, 22.38, 13.89. FT-IR: (neat) [cm^–1^] 2,200 (**−C≡N**, Nitril) Yield 87%. Khan et al. also reported the synthesis and NMR characterization of 2-hydroxyheptanenitrile in their work.^32^

##### Synthesis of 2-Hydroxyheptanamide (**10**)

Pure 2-hydroxyheptanenitrile **(9)** was reacted with H_2_O_2_ in DMSO and excess K_2_CO_3_. The reaction continued overnight by keeping the temperature below 10 °C. The resultant material was extracted with ethyl acetate, dried using a rotary evaporator, and crystallized from a mixture of ethyl acetate/hexane. ^1^H NMR (300 MHz, DMSO-*d*_6_) δ 7.09 (d, *J* = 19.6 Hz, 2H), 5.24 (s, 1H), 3.75 (dd, *J* = 7.4, 4.1 Hz, 1H), 1.59 (ddq, *J* = 15.2, 7.0, 3.6, 3.1 Hz, 1H), 1.53–1.34 (m, 1H), 1.38–1.15 (m, 6H), 0.86 (t, *J* = 6.6 Hz, 3H). ^13^C NMR (75 MHz, DMSO-*d*_6_) δ 177.03, 71.26, 34.68, 31.62, 24.79, 22.55, 14.40. ESI MS (+ve ionization): calcd for C_7_H_15_NO_2_ 145.20 observed 146.16. (M + H). FTIR: (neat) [cm^–1^] 1620(–C=O, amid). **Yield 90%**

##### Synthesis of 2-Hydroxyheptanoic acid (**11**)

Hydrolysis of the amide to carboxylic acid was done in an ethanol/water mixture by using an excess amount of NaOH (under reflux). Reaction progress was checked by TLC (MeOH/DCM)(5:95). Ethanol was evaporated under reduced pressure when most of the starting material was consumed. The resultant aqueous solution was washed (×3) with ether to remove the residual starting material (amide). Then, the mixture was acidified by using concentrated HCl. The carboxylic acid product was extracted by using ethyl acetate. The resultant yellowish solid was crystallized (×3) in ethyl acetate/hexane 1:4 to get a white crystalline material. ^1^H NMR (300 MHz, DMSO-*d*_6_) δ 3.90 (dd, *J* = 7.6, 4.6 Hz, 1H), 1.64–1.41 (m, 2H), 1.38–1.12 (m, 6H), 0.95–0.71 (t, 3H). ESI MS (negative ionization): calcd for C_7_H_15_N0_2_ 146 observed 145 (M – H), FT-IR: (neat) [cm^–1^] 1700 (–C=O, of carboxylic acid), 3200–3500 (OH of carboxylic acid) Yield 50%.

##### Synthesis of 5-Pentyl-1,3-dioxolane-2,4-dione (OCA of Heptanal) (**12**)

The hydroxyl acid was dissolved in extra dry THF, and activated charcoal was suspended in the solution. This mixture was purged with a nitrogen atmosphere. Triphosgene solution was added, followed by triethylamine. The reaction was continued for 72 h, and the mixture was filtered through filter paper. THF was then evaporated under reduced pressure, and the triethylammonium chloride salt was precipitated out with ether. The residual of the salt was extracted with ice water after filtration of the mixture through filter paper. The organic layer was dried quickly using Na_2_SO_4_. After evaporation of the ether, the resultant liquid material was dissolved into a minimal amount of hexane and crystallized out in −20 °C. ^1^H NMR (300 MHz,) δ 5.1–4.98 (m, 1H), 2.17–1.84 (m, 2H), 1.66–1.12 (m, 6H), 0.90 (q, *J* = 5.3 Hz, 3H). ^13^C NMR (75 MHz, Chloroform-*d*) δ: 167.12, 79.73, 30.82, 30.74, 23.60, 22.19, 13.81. FT-IR: (neat) [cm^–1^] 1790, 1820 (–C=O, oxazolidine-2,5-dione) Yield 74%

##### Ring-Opening Polymerization (**13**)

In a glovebag, a solution of 4-(Dimethylamino)pyridine (DMAP) in dry dichloromethane (DCM) (0.01 M in 2 mL) and freshly distilled isobutyl alcohol (IB, alcohol initiator, 0.01 M in 2 mL DCM) was added to 1 mmol of *O*-carboxy anhydride dissolved in 6 mL of DCM. The reaction mixture was stirred for 36 h under inert atmosphere. The reaction process releases CO_2_, which needs to be purged from time to time with a slow stream of nitrogen. The reaction progress was monitored by collecting the sample and checking the FT-IR spectra. DCM was concentrated under vacuum, when the polymerization reaction was over, and the polymer was precipitated in cold methanol.

## Results and Discussion

**Scheme 1:**
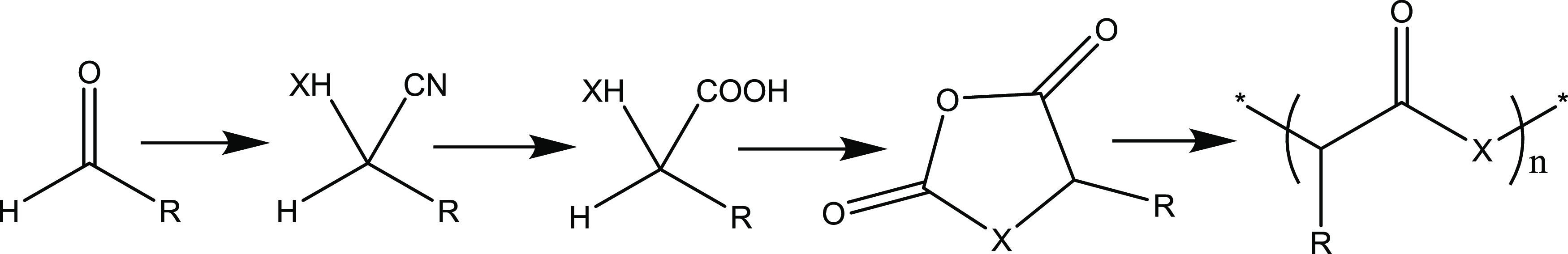
General Scheme for the Synthesis of α-Amino Acids and α-Hydroxy Acids from Aldehyde Using Strecker Synthesis

There is a growing demand for expanding the library of existing biodegradable polymers, particularly polypeptides and polyesters, owing to their biocompatibility and tunable physiochemical properties. To meet this demand, it is important to expand the library of existing building blocks of these polymers, i.e., α-amino acids and α-hydroxyl acids. For this purpose, we utilized the Strecker reaction, which is one of the oldest known methods for the synthesis of amino nitriles.^26−29^ It is also a simple and cost-effective method for the preparation of α-amino acids for both laboratory and industrial scales.^30^ This method allows conversion of different aldehydes including aliphatic, aromatic, or saccharide to amino/hydroxyl acids. Strecker amino acid synthesis involves treatment of aldehydes with ammonia and hydrogen cyanide (or equivalents like trimethylsilyl cyanide) followed by hydrolysis of the intermediate α-amino nitriles to α-amino acids (Scheme 1).

Using Strecker synthesis, we synthesized aliphatic amino acids and hydroxyl acids with hexanal as the starting material. These monomers were then polymerized to new “PLA-like” polypeptides and polyesters.

### Synthesis of α-Amino Acid from Hexanal and Its Polymerization to a New Aliphatic Polypeptide

**Scheme 2:**
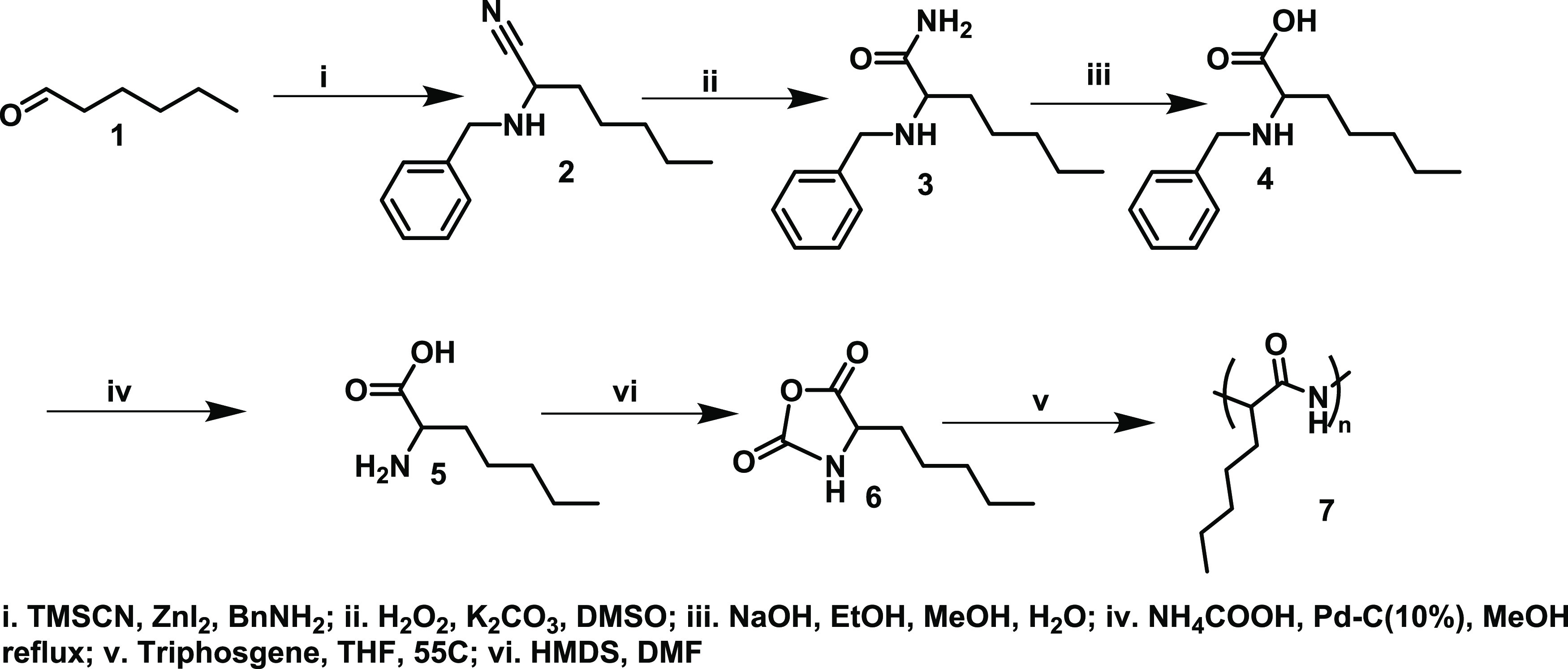
Synthesis of Heptane Amino Acid from Hexanal and Its Polymerization to Polypeptide

The conventional Strecker synthesis yields unstable amino nitrile, resulting in negligible and impure yield of acid/alkaline hydrolysis product. This is primarily due to the retro-Strecker reaction, in which α-amino nitriles convert into their corresponding imines during acid or alkaline hydrolysis.^33^ Davies et al.^34^ suggest using benzyl amine instead of free ammonia. Substituting free ammonia with benzyl amine provided a stable amino nitrile with a high yield. The resultant benzyl-protected amino nitrile undergoes a two-step hydrolysis process to yield the desired amino acid. Subsequently, the benzyl group was cleaved via hydrogenolysis to yield the desired free amino acid as described in Scheme 2.

In detail, hexanal (**1**) was reacted with trimethylsilyl cyanide (TMSCN) using ZnI_2_ as a catalyst. Benzyl amine was added to obtain the desired benzyl-protected α-amino nitrile of hexanal (**2**), confirmed by H NMR, showing aromatic protons at ∼8 ppm. Wiles et al. also reported the synthesis of α-amino nitrile of hexanal employing heterogeneously catalyzed Strecker reaction.^35^ The use of benzyl amine in this step provided a benzyl-protected amine, which is more stable compared to the free amine, and it can avoid the retro-Strecker reaction during subsequent alkaline hydrolysis of nitrile to carboxylic acid, a relatively harsh synthesis.

The hydrolysis of the α-amino nitrile to the corresponding carboxylic acid was carried out in a stepwise reaction, as described by Urogdi et al.^36^ The first step was conversion of the nitrile to amide by using hydrogen peroxide and K_2_CO_3_. This is a mild condition that involves the reaction of nitrile with an alkaline solution of hydrogen peroxide. The strongly nucleophilic hydrogen peroxide adds to the nitrile, and the resultant adduct gives the amide.

The amide obtained in the previous step was further hydrolyzed to a carboxylic acid in an aqueous alkaline solution. Schafer et al. also reported the synthesis of this material from its amide precursor via acid hydrolysis.^37^ However, they have reported yield of <30%. Using alkaline hydrolysis pure amino acid with a reasonable yield (40%) was isolated using this synthesis method. The next step of the synthesis was the deprotection of the benzyl group from the amine to obtain free amino acid. Deprotection was achieved by refluxing compound **4** in methanol, using NH_4_COO as a hydrogen-donating agent and activated palladium*/*charcoal (10% Pd) as a catalyst. The progress of the reaction was monitored using TLC (DCM/MeOH 95:5). There are several literature references to get the material, but none of them was not via the Strecker reaction methodology. Kokotos et al. chlorinated heptanal and oxidized it toward α-chloro acids. The amination was done by nucleophilic substitution.^38^ Tirrell et al. synthesize 5 by alkylation of diethyl acetamidomalonate with the appropriate tosylate.^39^ The resultant heptane amino acid (**5**) served as a starting material for the synthesis of the active *N*-carboxy anhydride precursor for the ring-opening polymerization. The ring-opening polymerization via *N*-carboxy anhydride formation is the most widely used method to synthesize amino acid homopolymers.^24^ The resultant amino acid (**5**) was then cyclized in the presence of triphosgene in ethyl acetate to form the activated intermediate *N*-carboxy anhydride (**6**).

**Figure 1:**
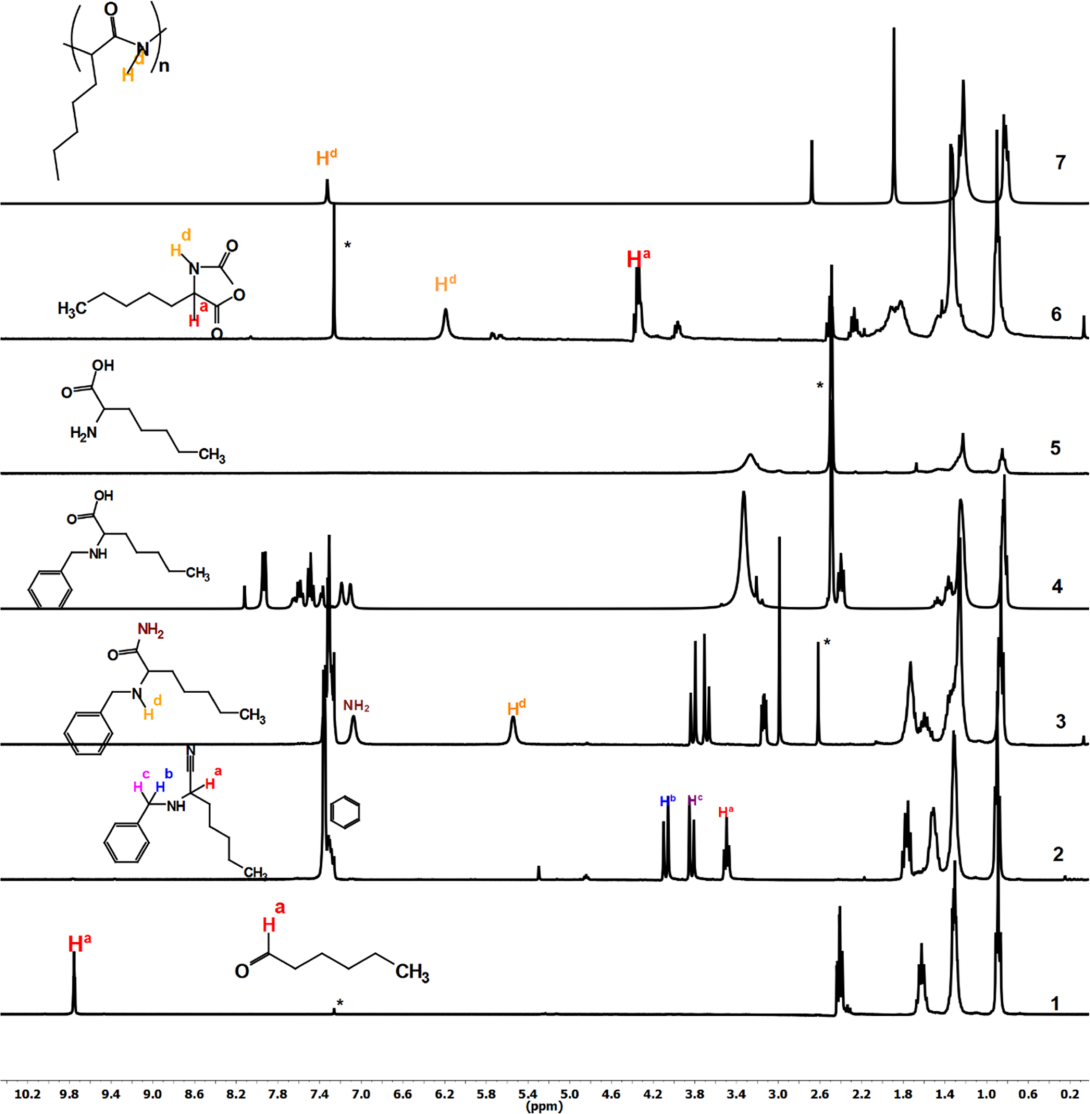
^1^H NMR analysis for polyamide synthesized from hexanal via Strecker amino acid synthesis.

Figure 1 depicts the ^1^H NMR analysis for the polyamide synthesized from hexanal. Amino nitrile was obtained by reacting the aldehyde with TMSCN and benzyl amine. In the resultant compound (2), the aldehyde proton disappeared and a new peak emerged at ∼3.6 ppm, corresponding to the proton α to the nitrile bond and marked as **H**^**a**^. Additionally, peaks corresponding to the aromatic protons of the benzyl group from the benzyl amine were also observed. Upon hydrolysis of the nitrile group, the formation of the amide (Compound **3**) led to the appearance of new amide protons in the NMR spectrum, which were eliminated after alkaline hydrolysis to obtain the desired amino acid **4**. The benzyl-protecting group was then removed from the amine to obtain the free amino acid **5**. The *N*-carboxy anhydride (NCA) **6** of the resultant amino acid was obtained by reacting it with triphosgene, which is a precursor for ROP polymerization.

### Synthesis and Analysis of the Resulting Aliphatic Polypeptide

**Figure 2:**
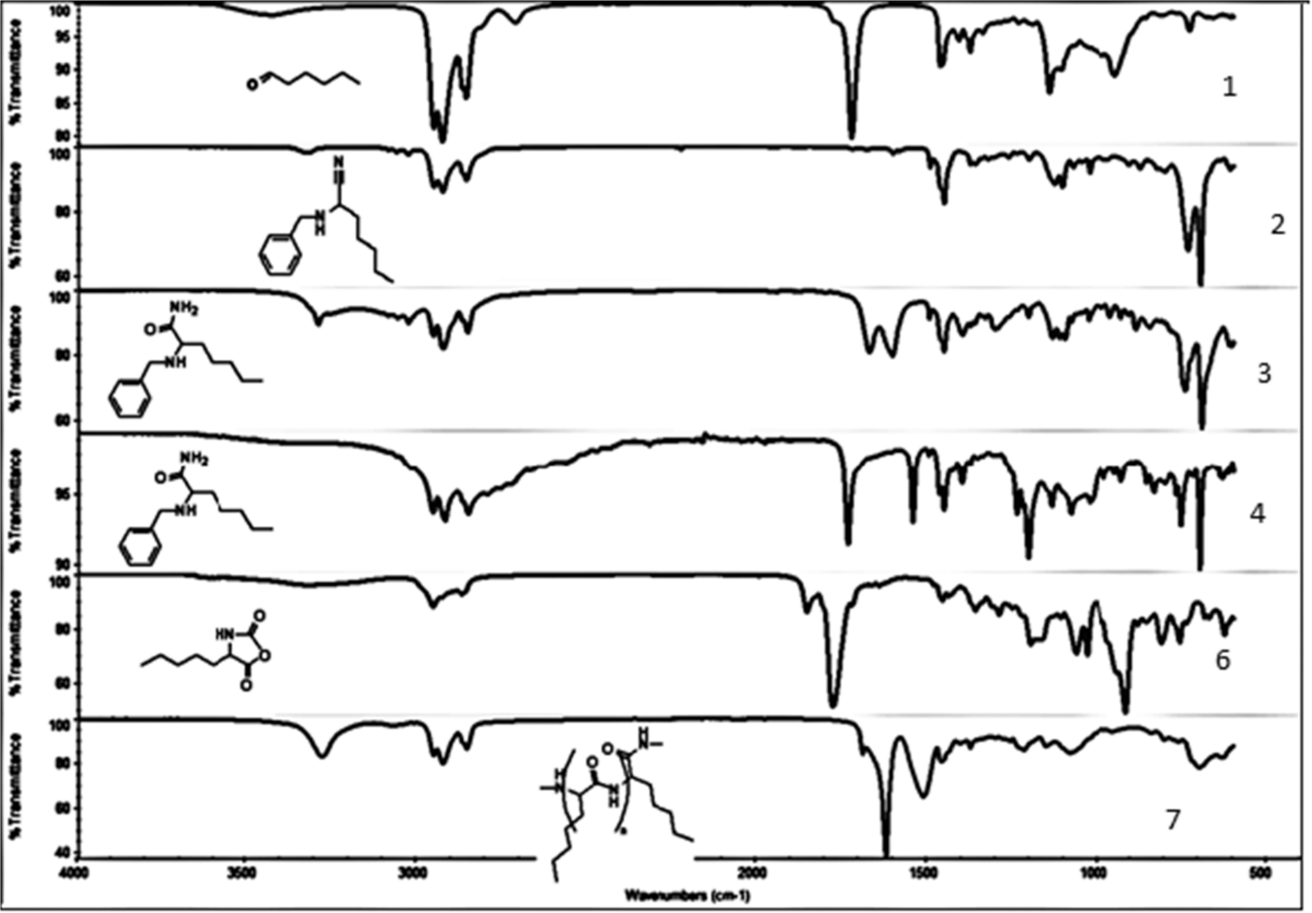
FT-IR analysis of the polypeptide synthesized from hexanal via Strecker synthesis.

For polymerization, hexamethyldisilazine (HMDS) was chosen as the initiator for the ring-opening polymerization of the *N*-carboxy anhydride monomer. HMDS-initiated polymerization is a controlled and validated strategy for the synthesis of amino acid homopolymers.^40^ Polymerization progress was monitored using FT-IR spectroscopy that shows the elimination of the anhydride carbonyl peak and emergence of the amide peak characteristic of the polypeptide. Figure 2 depicts the FT-IR analysis of the polypeptide synthesized. Upon reaction of the aldehyde with TMSCN, the carbonyl peak of the aldehyde disappeared and a new peak appeared at 2223 cm^–1^ corresponding to the nitrile functional group. Subsequent hydrolysis of the nitrile using H_2_O_2_ and K_2_CO_3_ resulted in the formation of the amide bond, indicated by a peak at 1672 cm^–1^. Further alkaline hydrolysis of the amide bond provided the desired carboxylic acid, observed at 1732 cm^–1^(corresponding to the –C=O acid). Following deprotection of the benzyl group, the resulting free amino acid was reacted with triphosgene to generate the desired *N*-carboxy anhydride, which was confirmed by peaks at 1790 and 1820 cm^–1^ (–C=O and oxazolidine-2,5-dione, respectively). The N-carboxy anhydride was then polymerized to form the polypeptide via ring-opening polymerization, as indicated by the peak at 1650 cm^–1^ (–C=O amide).

With the progress of the polymerization reaction, the polypeptide started to precipitate. The resultant polymer was purified by repeated precipitation in cold methanol to obtain a white powder. The polymer exhibited limited solubility, being insoluble in most solvents except for a slight solubility in DMSO. While ^1^H NMR was recorded for this polymer, GPC analysis could not be performed due to its limited solubility. The insolubility of this aliphatic polyamide can be attributed to the presence of several hydrogen bonds originating from the amide bonds within the polymer structure. In addition, the repeating units of this polymer are pendant aliphatic groups with long chains (heptane amino acid), which contribute to its hydrophobic nature and insolubility. Thermal analysis performed by DSC revealed that the polymer exhibited a crystalline structure with a high melting point (*T*_m_ = 199 °C).

### Synthesis of Polyester from Hexanal

As mentioned above, the primary goal of this research is to expand the existing library of α-amino and α-hydroxyl acids and thereby subsequent development of new polypeptide and polyester-based biomaterials. For this purpose, we chose Strecker amino acid synthesis and synthesized aliphatic polypeptides with the methodology described above. Using a similar strategy, we planned to synthesize α-hydroxy acids from hexanal. Our initial thought was to hydroxylate the amine group of the amino acid synthesized above and to polymerize the resulting hydroxyl acid to polyester.

First Hexanal was reacted with trimethylsilyl cyanide to yield α-silyloxynitrile. Subsequently, potassium fluoride was added to cleave the silyl-protecting group, resulting in the formation of hydroxyl nitrile **9**. The nitrile group was hydrolyzed first to amide followed by alkaline hydrolysis to obtain the desired hydroxyl acid. The hydroxyl acid was further cyclized to form *O*-carboxy anhydride, which was subsequently polymerized by ring-opening polymerization to produce the corresponding polyester. In the classic Strecker synthesis, amino nitriles are obtained by condensing aldehyde with trimethylsilyl cyanide in the presence of a catalytic amount of ZnI_2_. This process leads to the formation of an intermediate known as α-silyloxynitrile compound **(9)**,^41^ which is a pseudohydroxy nitrile whose hydroxyl group is protected with silyl ether. Generally, cleavage of silyl ethers is done using fluoride compounds, inorganic bases, and inorganic acids.^42^ Tetrabutylammonium fluoride (TBAF) is the most commonly used desilylating reagent. However, it is known to have possible side reactions caused by the nucleophilicity of the fluoride ion. Song et al.^43^ developed a mild and efficient protocol for the deprotection of silyl ethers using potassium fluoride in tetraethylene glycol.

According to this procedure, the ether groups of the tetraethylene glycol act as a Lewis base toward K^+^, “freeing” the counteranion (F^–^), as well as “enhancing” the solubility of the alkali metal salts. On the other hand, one of the two terminal hydroxyl groups of tetraethylene glycol forms controlled H-bonding with the fluoride anion, decreasing the basicity of the nucleophile. The other OH group can simultaneously activate the electrophile by hydrogen bonding, thereby stabilizing the transition state. This procedure provided the desired hydroxynitrile **(10)** with a good yield.

Once α-hydroxyl nitrile was obtained, it serves as a readily available precursor for the synthesis of α-hydroxyl acid. Therefore, we employed the same two-step hydrolysis method used for the amino acid to obtain the desired α-hydroxy acid **(11)**. Dennig et al. also reported biocatalytic amination of carboxylic acids to synthesize α-amino acids.^44^

### Synthesis and Characterization of Polyester

Ring-opening polymerization of the 5-membered active precursor, *O*-carboxy anhydride, is a well-established and efficient method for the polymerization of α-hydroxy acids. This polymerization occurs under mild conditions and in a controlled fashion.^22^ Hence, prior to polymerization, the resulting hydroxyl acid **(11)** was reacted with triphosgene to obtain the *O*-carboxy anhydride precursor **(12)**.

The polymerization rate and resultant molecular weight are controlled by the amount of the alcohol initiator and catalyst used. The most commonly used alcohol initiator is isobutanol, while the most commonly used catalyst is 4-(Dimethylamino)pyridine. Hence, the polymerization of *O*-carboxy anhydride was carried out using isobutanol as the initiator and D-MAP as the catalyst. When the reaction was completed, a pasty brownish ointment-like polyester **(13)** was obtained. Molecular weight analysis showed that the resultant polymer has a molecular weight of ∼3000 Da relative to polystyrene standard. DSC analysis showed that the polymer is amorphous with a glass transition temperature of 35 °C. The amorphous nature of this polymer can be attributed to two factors. When aldehyde is converted to hydroxy acid via Strecker reaction, the resultant product is a racemic hydroxyl acid. As a result, an isotactic/syndiotactic polymer is obtained. Furthermore, the resulting polyheptane ester has a long pendant aliphatic side group which interferes with the 3D arrangement of the polymer and contributes to its amorphous nature.

**Figure 3:**
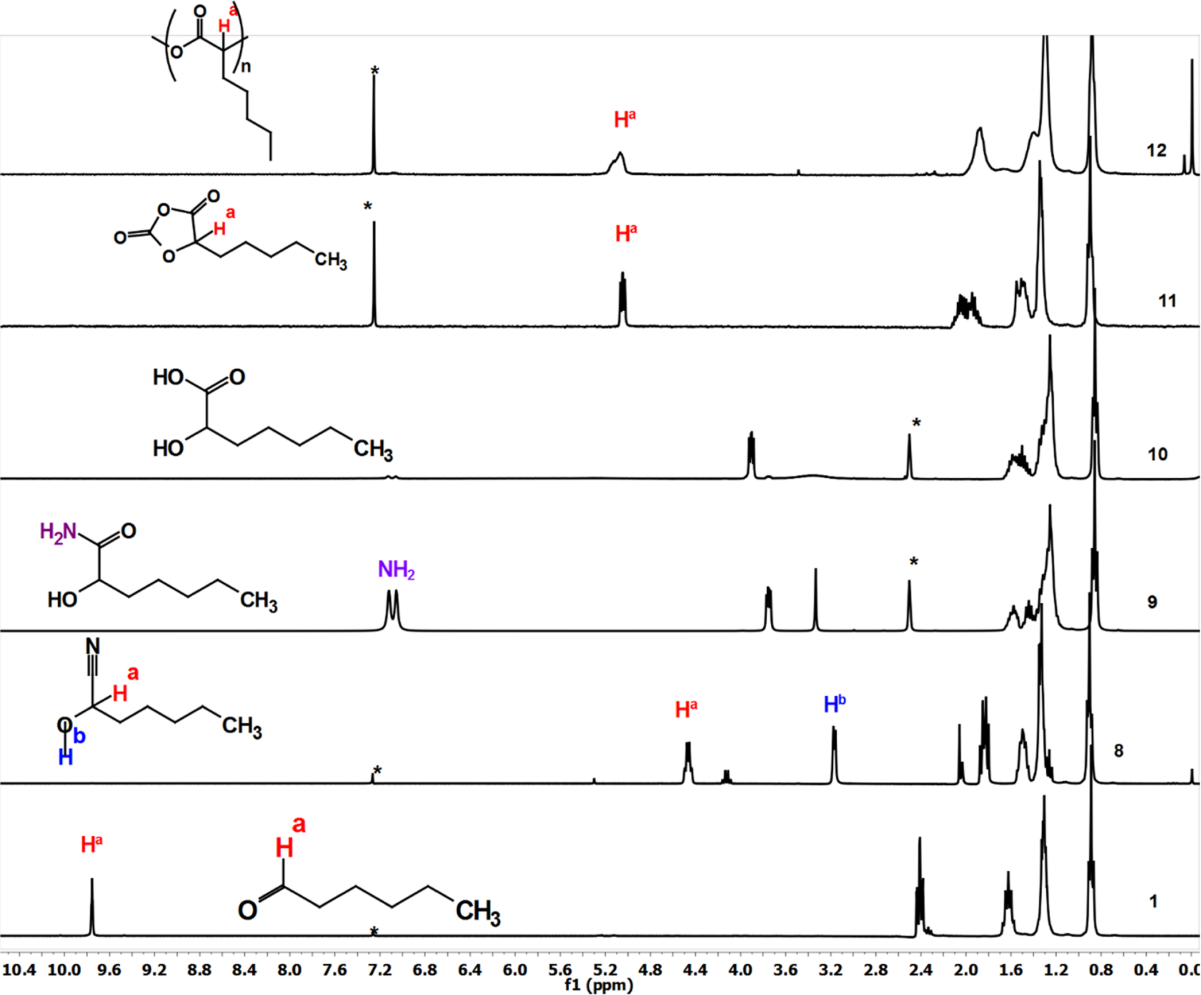
^1^H NMR analysis for the synthesis of polyester starting from hexanal.

Polyester synthesis was characterized by employing NMR and FTIR. Figure 3 depicts the NMR spectra of the polyester synthesized. The aldehyde proton (marked as **H**^**a**^) at ∼9.6 ppm was no longer observed after reacting it with trimethylsilyl cyanide. Instead, a new peak observed at ∼6.4 ppm corresponds to the proton α to the nitrile group. Hydrolysis of the nitrile via H_2_O_2_ and K_2_CO_3_ converted it into an amide (**9**) (marked as **NH**_**2**_ at ∼7.2 ppm). The alkaline hydrolysis of the amide yielded the desired α-hydroxyl acid, which was subsequently cyclized to *O*-carboxy anhydride, serving as a precursor for the ring-opening polymerization.

**Figure 4:**
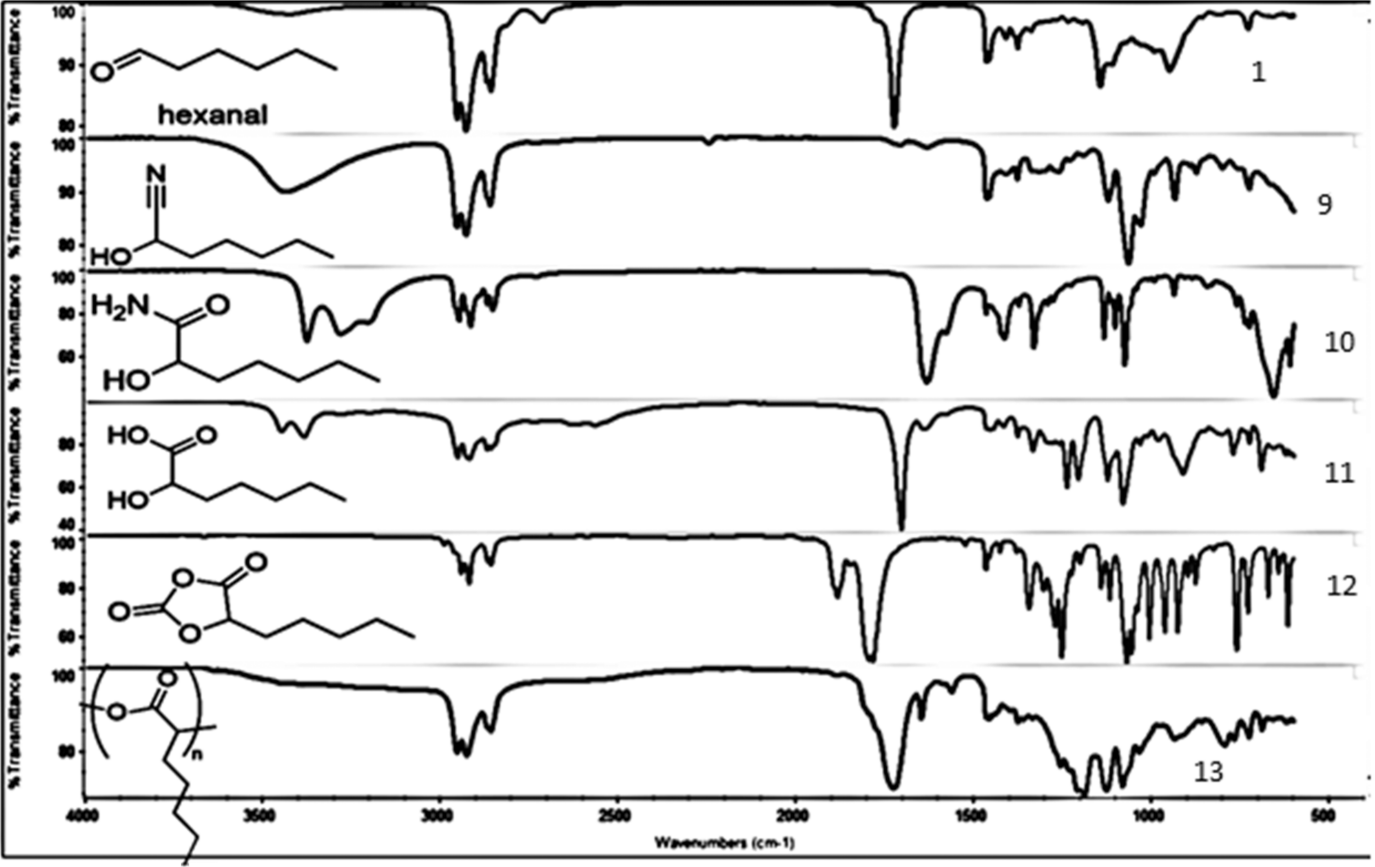
FT-IR analysis of the polyester synthesized from hexanal.

Figure 4 depicts the NMR spectra of the polyester synthesized. Following the reaction of aldehyde with TMSCN, the carbonyl peak of the aldehyde was eliminated and the nitrile peak at 2223 cm^–1^ evolved. Hydrolysis of nitrile using H_2_O_2_ and K_2_CO_3_ resulted in the formation of the amide bond, observed at 1672 cm^–1^. Alkaline hydrolysis of the amide bond provided the desired carboxylic acid, evident by the peak at 1732 cm^–1^(–C=O acid). The resulting hydroxyl acid was reacted with triphosgene to obtain the desired *O*-carboxy anhydride, indicated by the peaks at 1808 and 1888 cm^–1^ (C=O anhydride). The OCA precursor was subsequently polymerized to polyester, as indicated by the presence of the –C=O ester peak at 1730 cm^–1^.

## Conclusions

In this study, we successfully synthesized α-amino and α-hydroxy acids from hexanal as a model aldehyde using a modified Strecker synthesis approach. By using benzyl amine as the amine-donating agent instead of free ammonia, the amino acid was polymerized into an aliphatic polypeptide via ring-opening polymerization of the *N*-carboxy anhydride derivative. Additionally, the α-hydroxy acid was synthesized from the reaction of hexanal with TMSCN to form a trimethylsilyl-protected hydroxynitrile. The silyl group was cleaved using potassium fluoride with tetraethylene glycol. The resultant hydroxynitrile was hydrolyzed to hydroxyl acid and subsequently polymerized to polyester via ring-opening polymerization. This strategy opens up new possibilities for converting not only aliphatic aldehydes but also saccharides into hydroxyl acid/amino acids. By strategically protecting the multihydroxyl groups with appropriate groups, like benzyl ether, before functionalization reaction, we have expanded the range of molecules that can be transformed. The current strategy can be employed further for the synthesis of a series of polypeptides and poly(α-hydroxy esters) library for their application in biomaterial science like drug delivery and tissue engineering applications. We plan to explore this prospect further in our forthcoming studies.
